# Visual and auditory attention in individuals with *DYRK1A* and *SCN2A* disruptive variants

**DOI:** 10.1002/aur.3202

**Published:** 2024-07-30

**Authors:** Caitlin M. Hudac, Kelsey Dommer, Monique Mahony, Trent D. DesChamps, Brianna Cairney, Rachel Earl, Evangeline C. Kurtz‐Nelson, Jessica Bradshaw, Raphael A. Bernier, Evan E. Eichler, Emily Neuhaus, Sara Jane Webb, Frederick Shic

**Affiliations:** ^1^ Department of Psychology University of South Carolina Columbia South Carolina USA; ^2^ Center for Autism and Neurodevelopment (CAN) Research Center University of South Carolina Columbia South Carolina USA; ^3^ Institute for Mind and Brain University of South Carolina Columbia South Carolina USA; ^4^ Center for Child Health, Behavior and Development Seattle Children's Research Institute Seattle Washington USA; ^5^ Department of Psychiatry and Behavioral Sciences University of Washington Seattle Washington USA; ^6^ Department of Pediatrics Indiana University Indianapolis Indiana USA; ^7^ Department of Genome Sciences University of Washington Seattle Washington USA; ^8^ Howard Hughes Medical Institute University of Washington Seattle Washington USA; ^9^ Department of Pediatrics University of Washington Seattle Washington USA

**Keywords:** attention, autism spectrum disorder (ASD), conversational flow, DYRK1A, electroencephalography (EEG), eye tracking, genetic etiology, SCN2A, social attention

## Abstract

This preliminary study sought to assess biomarkers of attention using electroencephalography (EEG) and eye tracking in two ultra‐rare monogenic populations associated with autism spectrum disorder (ASD). Relative to idiopathic ASD (*n* = 12) and neurotypical comparison (*n* = 49) groups, divergent attention profiles were observed for the monogenic groups, such that individuals with DYRK1A (*n* = 9) exhibited diminished auditory attention condition differences during an oddball EEG paradigm whereas individuals with SCN2A (*n* = 5) exhibited diminished visual attention condition differences noted by eye gaze tracking when viewing social interactions. Findings provide initial support for alignment of auditory and visual attention markers in idiopathic ASD and neurotypical development but not monogenic groups. These results support ongoing efforts to develop translational ASD biomarkers within the attention domain.

## Lay Summary

This study tested auditory and visual attention in children, adolescents, and young adults with and without a known genetic change that is related to autism. Autistic people without a known genetic change and non‐autistic people: (1) had larger brain responses to new sounds compared with repeated sounds and (2) looked more at the speaker when they looked directly at the camera compared with looking at other people sitting at a table. People with DYRK1A genetic changes had different auditory attention whereas people with SCN2A genetic changes had different visual attention.

## INTRODUCTION

There is a growing, urgent need to quantify biological indicators or markers (herein, “biomarkers”) that are unique to autism spectrum disorder (ASD). In part, disentangling how biological mechanisms relate to neural, behavioral, and clinical phenotypes of ASD may propel us toward precision medicine and targeted treatment (Loth et al., 2016). Despite the relevance of biomarkers to facilitate drug, medical, and diagnostic device approval (Mattes & Goodsaid, 2018), ASD biomarker development has been stunted by the extensive phenotypic heterogeneity that exists within ASD (Kim et al., 2016; Tillmann et al., 2020). Evaluating biomarkers solely in populations ascertained based upon clinical diagnosis may reduce the likelihood of identifying shared underlying biological mechanisms. In contrast, a genetics‐first approach that examines phenotypes of autistic individuals with ultra‐rare disruptive variants in the same gene (Consortium, 2012; Stessman et al., 2014) may serve as an important way to accelerate development of biomarkers and identify mechanistic target engagement metrics for clinical trials. Genetic etiologies of ASD, including de novo disruptive single gene mutations and copy number variations, may account for upwards of 25% of ASD cases (Iossifov et al., 2012, 2014; Kaufman et al., 2010; McCarthy et al., 2014).

As such, biomarker development must be inclusive of monogenic populations and have a translational link between animal and human models (Ewen et al., 2019; Sahin et al., 2018). Attention may serve as an optimal biomarker, considering that attention signals can be measured readily across species (Campbell et al., 2014). At its basis, attention describes how a person mentally selects a target to guide their behavior (Wu, 2024), encompassing low‐level phenomena such as early perceptual processing (Johnston & Wilson, 1980; Sanders & Astheimer, 2008) and higher level phenomena such as gating mechanisms that regulate cognitive functions (Cohen et al., 2020; Noppeney, 2021; Wiesman & Wilson, 2020). Early theories of ASD (see Allen & Courchesne, 2001) suggested that differences in the attention system may contribute to atypical social‐cognitive skills that are observed in ASD (APA, 2013). In other words, if broader, generalized attention is disrupted in ASD—whether inside or outside of a social context—this may disrupt social opportunities and lead to behavioral differences observed in ASD. Indeed, there is evidence of broad attention disruptions in ASD, including clinical reports that suggest between 31% and 95% of autistic individuals exhibit broader symptoms of inattention and/or hyperactivity and impulsivity and have elevated rates of co‐occurring attention deficit hyperactivity disorder (ADHD) (Antshel et al., 2013, 2016; Reiersen & Todd, 2008). However, it is critical to evaluate the role of social attention (i.e., attention directed toward social information) in ASD (Dawson et al., 2004; Falck‐Ytter et al., 2023). Indeed, social attention has been a key focus of biomarker development in consortia worldwide (Loth et al., 2017; McPartland et al., 2020; Oakley et al., 2022), suggesting unique social attention patterns in autistic participants (Shic et al., 2023; Webb et al., 2023). However, monogenic populations are often excluded from large‐scale biomarker development, despite observing attention‐related clinical concerns in monogenic populations and the growing need to evaluate biomarkers in tandem with basic, translational, and animal‐based science (Hudac & Webb, 2024).

In sum, neither general attention nor social attention is well‐characterized using biologically derived or cognitive neuroscience methods. Here, we focus on two ultra‐rare monogenic groups (DYRK1A and SCN2A) as a preliminary first step to characterize biomarkers of attention mechanisms in rare genetic subgroups of ASD. First, DYRK1A, dual‐specificity tyrosine‐(Y)‐phosphorylation‐regulated kinase 1 A, is located within the Down syndrome critical region on chromosome 21. DYRK1A is responsible for neural proliferation and neurogenesis, as well as synaptic regulation and neural aging (Krumm et al., 2014; Tejedor et al., 1995), all critical processes for attention. Disruptive DYRK1A variants are known to account for 0.1%–0.5% of individuals with intellectual disability and/or ASD (van Bon et al., 2015; Bon et al., 2016; Courcet et al., 2012; Fitzgerald et al., 2015; O'Roak et al., 2012). ASD diagnoses are reported in 43% (18/42) of DYRK1A carriers, with ASD symptoms observed in 69% (42/61) of carriers, although social motivation is a noted strength (Earl et al., 2017). Second, SCN2A encodes the voltage‐gated sodium channel Na_v_1.2 that serves a major role in synaptic depolarization and action potential initiation within early development (Sanders et al., 2018; Yamagata et al., 2017) and later, persistent role for postsynaptic regulation of excitatory synapses (Spratt et al., 2019). Primary phenotype of SCN2A varies based upon genetic variant, such that gain‐of‐function variants potentiate glutamatergic neuronal excitability that is linked to infantile‐onset seizure phenotypes, whereas loss‐of‐function variants impair neuronal excitability and are linked to neurodevelopmental phenotypes including ASD (Sanders et al., 2018). Disruptive SCN2A variants are known to account for 0.1%–0.5% of individuals with intellectual disability and/or ASD (Ben‐Shalom et al., 2017; Reynolds et al., 2020).

From a clinical standpoint, these groups were chosen due to exhibit‐increased rates of ADHD and known attention problems. Attention problems are variably reported in DYRK1A, with ~20%–45% diagnosed with ADHD or have parent reported symptoms consistent with ADHD (Durand et al., 2022; Ruaud et al., 2015). In addition, hyperactivity is implicated in 33% of DYRK1A carriers (Earl et al., 2017) and heightened sensory sensitivity is found in DYRK1A at rates above that observed in ASD without a known genetic etiology (i.e., idiopathic ASD; herein, idiopathic autism spectrum disorder [iASD]) (Hudac et al., 2023). In addition, anxious behaviors are common in 27% of DYRK1A carriers, with notable social anxiety observed as a part of the clinical DYRK1A phenotype (Earl et al., 2017). Regarding SCN2A, (Ben‐Shalom et al., 2017; Reynolds et al., 2020; Sanders et al., 2018; Spratt et al., 2019; Yamagata et al., 2017) case studies report ADHD and poor attention on standardized neuropsychological measures (Dhamija et al., 2013; Mangano et al., 2022). Genetic studies of ADHD have also implicated SCN2A (Peyre et al., 2024), suggesting it is increasingly important to know markers of attention in SCN2A. Basic and nonhuman work is investigating attention disruption as a possible mechanism of the SCN2A phenotype, including visual subcortical processes (Wang et al., 2023) and other pathways involved in axonal excitability (Indumathy et al., 2021).

### Current study objectives

Aligned with other large‐scale efforts (Loth et al., 2017; McPartland et al., 2020), we utilized existing eye tracking and electroencephalography (EEG) biomarker paradigms to capture two different modalities of attention. We examined both (a) social attention biomarkers using a visual attention eye‐tracking task with the hypothesis that people look more to someone who is speaking and looking toward you and (b) broad/generalized attention biomarkers using an auditory attention EEG task with the hypothesis that the attention orienting P3a amplitude response will be larger to novel than redundant auditory sounds. To better understand the potential unique phenotypes in the monogenic group, we examine these markers relative to an idiopathic ASD group without a known genetic etiology and neurotypical controls. As the first exploratory study in this area, our objective was to examine whether these attention biomarkers derived from individuals with DYRK1A and SCN2A are distinct from neurotypical and iASD comparison groups to provide preliminary evidence of unique phenotypes.

## MATERIALS AND METHODS

### Participants

Demographic data are provided in Table 1. Genetic characterization is available in Table S1. Data were combined across three studies as follows. First, participants with known disruptive genetics variants were enrolled in genetics first behavioral phenotyping studies (Grant # R01 MH101221; 2019 Action Potential Grant), which involves deep‐phenotyping of probands with the known variant and their biological parents. We focused this study on DYRK1A and SCN2A because these two genetic groups held in‐person family and scientific meetings that aided and facilitated participation in this study. Thus, here, we include nine DYRK1A and five SCN2A participants aged 2–18 years who completed separate eye‐tracking and EEG batteries. An additional two DYRK1A and three SCN2A participants were excluded due to poor initial eye‐tracking calibration (i.e., thus unable to validate gaze), and one DYRK1A participant did not have sufficient EEG data (<20 trials per condition). Second, two comparison groups were drawn from another study (Grant # R01 MH100047) that used the same tasks: first, an idiopathic ASD group (iASD; *n* = 12) with a research‐confirmed ASD diagnosis and no known genetic variant associated with ASD as confirmed by exome sequencing (Stessman et al., 2017); and second, a neurotypical group (NT; *n* = 49) that were included due a lack of ASD diagnosis and symptoms based upon parent report. This is the first manuscript to report the eye‐tracking data; EEG data from the comparison groups were previously published (Hudac et al., 2018).

**TABLE 1:** Sample characteristics.

	DYRK1A	SCN2A	iASD	NT	*p*‐value	Direction of significant group effects
Sex M:F	6:3	3:2	7:5	27:22	0.932	
Age in years *M* (SD)	9.73 (5.88)	9.5 (7.74)	11.17 (3.46)	8.74 (4.16)	0.425	
Age in years range	4–19	2–21	8–18	4–18		
NVIQ *M* (SD)	48 (18.83)	35.2 (33.55)	97.58 (11.08)	116.53 (14.15)	<0.001	DYRK1A, SCN2A<iASD<NT
NVIQ range	23–79	7–89	79–109	85–153		
VIQ *M* (SD)	45.11 (16.6)	38.8 (40.58)	107.92 (12.12)	115.53 (14.74)	<0.001	DYRK1A, SCN2A<iASD<NT
VIQ range	17–64	6–85	88–137	80–153		
ADOS‐2 CSS *M* (SD)	7.78 (2.11)	7 (2.16)	7.09 (2.43)	n/a	0.886	
ADOS‐2 CSS range	5–10	5–10	2–10	n/a		
Social affect *M* (SD)	6.56 (2.83)	6.75 (2.22)	8.22 (3.31)	n/a	0.470	
RRBs *M* (SD)	8.56 (1.88)	7.75 (1.71)	5.44 (3.4)	n/a	0.057	
VABS composite *M* (SD)	56.75 (6.41)	57 (24.34)	74.42 (7.19)	n/a	0.004	DYRK1A, SCN2A<iASD
VABS composite range	48–69	25–84	65–85	n/a		
Communication	24 (4.72)	17.4 (9.02)	31.5 (5.81)	n/a	<0.001	DYRK1A, SCN2A<iASD
Socialization	22.25 (8.91)	23.2 (11.08)	31.33 (4.81)	n/a	0.0322	DYRK1A<iASD
Valid EEG overall	73.6%	63.2%	80.8%	84.0%	<0.001	SCN2A<iASD, NT
Frequent % of 188 trials	70.4%	60.8%	80.3%	81.7%	0.02	SCN2A<NT
Novel % of 72 trials	76.7%	65.6%	81.3%	86.3%	0.06	
Valid ET overall	87.0%	81.6%	92.3%	94.1%	<0.001	DYRK1A, SCN2A<iASD<NT
Conversation % of 48 s	82.0%	80.3%	91.2%	93.8%	<0.001	DYRK1A, SCN2A<iASD<NT
Dyadic bid % of 48 s	92.0%	83.0%	93.4%	94.4%	<0.001	SCN2A<iASD<NT

*Note*: IQ scores unavailable from 1 SCN2A and 1 iASD participant. VABS unavailable for 1 DYRK1A participant.

Abbreviations: ADOS‐2 CSS, Autism Diagnostic Observation Schedule, Revised clinical severity score; EEG, electroencephalography; ET, eye tracking; iASD, idiopathic autism spectrum disorder; *M*, mean; n/a, not available; NT, neurotypical; NVIQ, nonverbal intelligence quotient; SD, standard deviation; VABS, Vineland Adaptive Scales.

All NT participants received assessments of cognitive functioning (see Table 1). All other participants received a comprehensive diagnostic evaluation consisting of measures of cognition (WASI‐II [Weschler, 2011] or DAS‐II [Elliott, 2007]), adaptive skills (Vineland Adaptive Behavior Scale‐II, VABS, [Sparrow et al., 2005]), and autism symptoms (Autism Diagnostic Observation Scheduled‐II, ADOS‐2 [Lord et al., 2012]; Autism Diagnostic Interview‐revised, ADI‐R, [Rutter et al., 1994]). Trained and research‐reliable clinicians administered all measures. These measures informed that clinical impressions and diagnoses were assigned based on DSM‐5 criteria, resulting in 25 participants with ASD (*n* = 12 iASD, *n* = 8 DRYK1A, *n* = 5 with SCN2A) and 13 participants with global developmental delay or intellectual disability (*n* = 8 DRYK1A, *n* = 5 SCN2A). Of note, there were no differences on ADOS‐2 clinical severity score, showcasing similar levels of autistic features across iASD and monogenic groups. It is important to note that none of the SCN2A participants in our sample experienced infantile seizures (before first year of life) that are indicative of seizure‐related phenotypes with gain‐of‐function effects of SCN2A excitability (Sanders et al., 2018). However, one SCN2A participant experienced seizure onset of 13 months; together with other functional measures, authors suggest mixed effects of SCN2A (Sanders et al., 2018).

No hearing or vision impairments were noted by parents or research staff for any participant and confirmed by medical examination for DYRK1A, SCN2A, and iASD participants.

### Eye‐tracking equipment, procedures, and analysis

Participants were seated in a quiet, dimly lit room, with their eyes level with approximately 650 mm distant from the center of a 24″ 1920 × 1200 pixel LED monitor. Gaze was tracked via an EyeLink 1000 Plus eye tracker (SR Research, Ottawa, Ontario, Canada) recording at 500 Hz. Stimuli were controlled and presented using Neurobehavioral Systems Presentation 18.1. Engaging, child‐friendly animated movies were first presented to attract participants' attention during setup. Following 5‐point calibration and validation, participants viewed a series of videos that made up a battery of interleaved social attention eye‐tracking tasks with periodic calibration validations presented throughout the session. Behavioral assistants focused on the participant and provided verbal redirections back to the screen when necessary, while an experimenter controlled the eye tracking and the stimulus presentation computers. Participants were asked to sit quietly and watch the screen. The total eye‐tracking session was 8–10 min.

**FIGURE 1:**
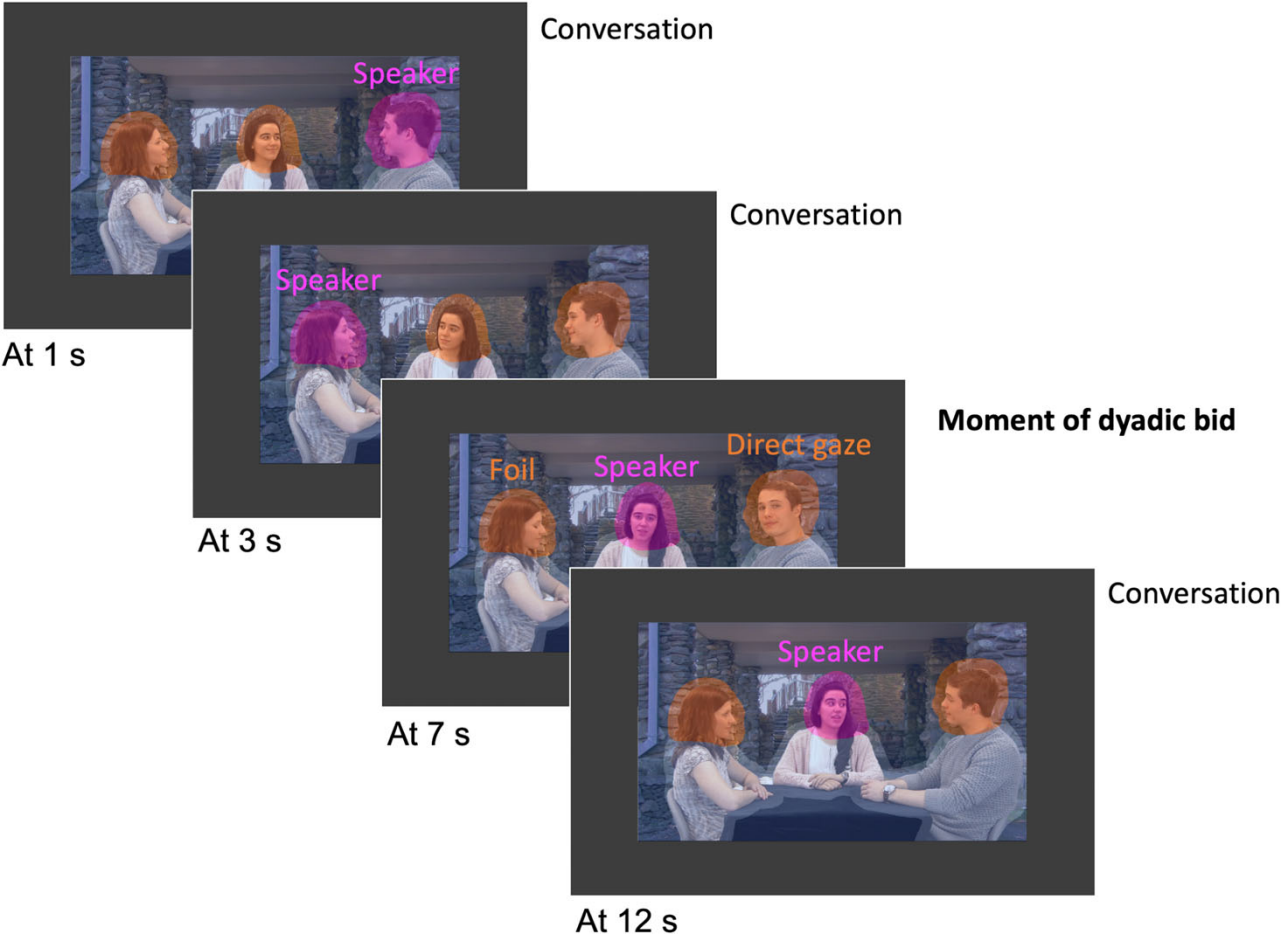
Example of eye‐tracking social attention stimuli. In each 16‐s video clip, three adults are in conversation together. At different times in each clip, the speaker looks directly to the camera during a moment of dyadic bid, while one nonspeaker looks down at the table (foil control) and the other nonspeaker looks directly to the camera (direct gaze control). Regions of interest were manually drawn around each person's head. In this analysis, all other areas were considered extraneous.

In the current study, we examined attention during a task targeting conversational flow and dyadic bids of attention that was interleaved with other videos and pictures (Figure 1). In this task, three actors are seated at a table and engage in conversations during a series of six 16 s video clips (total task time = 1.5 min). In each clip, each actor takes a single turn speaking in short sentences to the other two actors (e.g., “If it gets cold enough outside, we can go ice skating”), with the spoken‐to actors looking back at the speaker (conversational flow). Once during each clip, the speaker who just spoke or will speak to others next turns to look and speak directly to camera (i.e., “speaking” to the viewer) (dyadic bid). Here, we compare looking behaviors during two conditions: first, periods of conversational flow in which actors look at each other and take turns speaking; second, dyadic bids in which one actor looks and speaks directly to camera (i.e., “speaking” to the viewer). To serve as experimental controls and distractors during the dyadic bid, one nonspeaking actor also looks directly to camera (direct gaze control) and the other nonspeaking actor looks down at their hands while making subtle hand movements (e.g., adjusting watch; foil control).

Data were processed consistent with Shic et al. (2022). Trials were excluded when uncertainty regarding point‐of‐gaze was greater than 2.5° (1°= 42 pixels) or when less than 50% of eye‐tracking data were obtained (see Table 1 for data retention group averages). Because actors in trials moved minimally, all frame for the stimulus in each trial were averaged to form a single, representative image of the actors' positioning during the trial, and regions of interest were manually drawn over this representative image for each stimuli clip. Here, we focused on the speaker's head, the two nonspeaking actors' heads, and extraneous areas of the scene (i.e., background, bodies, and activity). Eye‐tracking outcome variables for each trial included the percentage of looking time toward each region of interest, derived by summing the total time gaze (i.e., the time‐varying spatial locations to which a participant's eyes appear to be directed) that was detected as directed to a region of interest, and dividing by the total time gaze was detected as being directed to any place on the presentation screen, and multiplying by 100 (Shic et al., 2022). Acquired eye‐tracking data streams were not processed into saccades and fixations due to interpretative ambiguities associated with fixation/saccade classification algorithms (refer papers below); instead, gaze durations (looking times) were computed as the total number of detected eye‐tracking samples within regions of interest multiplied by the eye‐tracking sampling rate (Shic et al., 2022). We also examined the number of gaze transitions between regions of interest relative to the overall amount of valid data as an indicator of region transition rate. We predicted that gaze will be directed toward the speaker's head, particularly during moments of dyadic bid relative to conversation.

### EEG equipment, procedures, and analysis

EEG methods were identical to our prior work (Hudac et al., 2018). In brief, EEG was recorded at 1000 Hz using a 128‐channel geodesic net (Magstim‐EGI, Eden Prairie, MN, USA) during an auditory oddball task in which participants attended to randomly presented frequent tones (70% of trials; 376 trials), infrequent tones (15% of trials; 72 trials), and novel sounds (e.g., chimes, chirps, drums; 15% of trials; 72 trials). Sinusoid tone stimuli (1000 and 750 Hz) were counterbalanced to frequent and infrequent conditions between subjects. Trials (520 total) were each 210 ms with a random intertrial interval jittered between 550 and 565 ms, and presented at 65 dB from Nata 3D speakers positioned 75 cm from the participant. Participants were instructed to silently watch a video of zoo scenes. Total task duration was 8 min.

Standard EEG processing consisted of offline filtering (0.1–40 Hz), segmentation from 100 ms prestimulus onset to 600 ms poststimulus onset, automatic then manually verified artifact detection based upon eye blinks and signals ≥140 μV, segment rejection, bad channel replacement, average re‐referencing, and baseline correction. Here, we focus on the novelty P3a peak amplitude as the maximum between 100 and 350 ms for the central medial electrodes, extracted from single trials (i.e., trials without artifacts) with the prediction that P3a amplitude will be larger to novel sounds relative to frequent tones. The P3a time window has previously been adopted for ages 4–23 years, and peak P3a was confirmed in this window for two participants in this study younger than 4 years.

### Statistical analyses

Similar single‐trial analytic strategies were employed for both eye tracking and EEG analyses. Linear mixed model analyses were generated separately for each outcome variable controlling for nonverbal IQ score and chronological age in months. Eye‐tracking outcome variables were percentage of looking at each spatial region of interest (3: speaker's head, nonspeakers head, and extraneous areas) and the number of gaze transitions (normed based upon the duration of valid data). EEG outcome variable was peak amplitude of the novelty P3a component. Fixed effects were group (4: DYRK1A, SCN2A, iASD, NT), condition (eye tracking 2: conversational flow, dyadic bid; or, EEG 2: frequent, novel), and group × condition interaction with covariates of nonverbal IQ and chronological age. Random intercepts modeled repeated trials for each participant.

Because of our objective to understand attention in the context of condition, we report condition effects for each group from each model and illustrate person‐level patterns where possible. Post hoc comparisons used Satterthwaite approximation to account for degrees of freedom and Bonferroni correction to address multiple comparisons.

As exploratory characterization, we sought to determine whether both methods elicit similar attention effects (i.e., condition differences). Thus, the extent of alignment between eye tracking and EEG biomarkers was assessed by establishing number of participants in each group with observed condition effects: (1) P3a amplitude larger for novel relative to frequent condition and (2) looking more to speaker during dyadic relative to conversation.

## RESULTS

### Visual social attention

**FIGURE 2:**
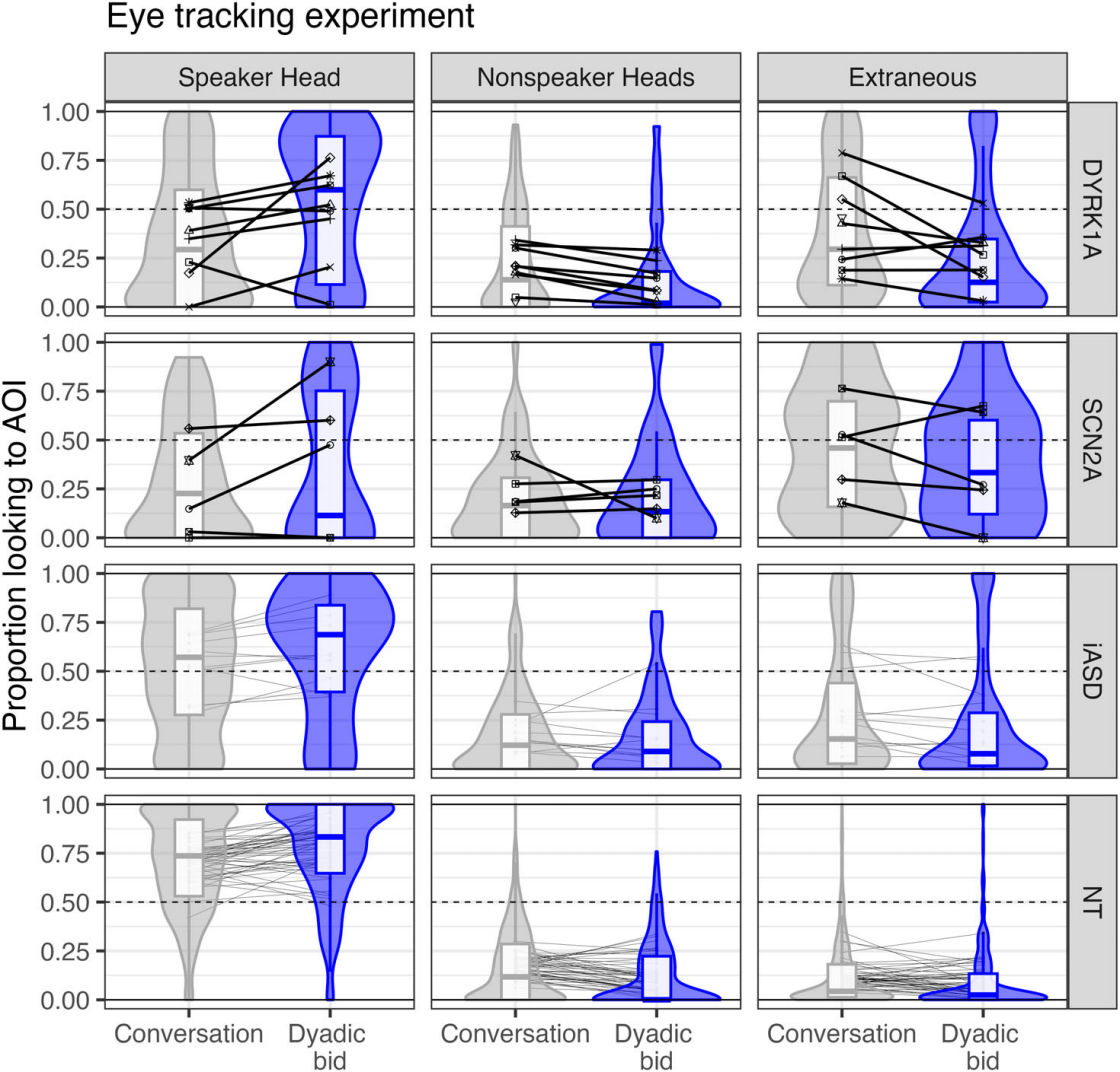
Proportion of looking during the dyadic bid eye‐tracking experiment. Group distributions are presented as violin plots to reflect group density and boxplots to indicate median (horizontal line in box) and inner quartiles (box). Proportion scores are presented as a line across condition for each individual, with marker shape indicating subjects within DYRK1A and SCN2A groups to orient to individual performance across region of interest. AOI, areas (region) of interest; iASD, idiopathic autism spectrum disorder; NT, neurotypical development.

Group‐level dwell time condition effects are illustrated in Figure 2 for each region of interest. Results are reported in Table 2, and trends are reported in Data S1. Regarding control variables, age did not impact visual social attention. Individuals with lower nonverbal IQ scores had less valid data and looked less at the speaker's head.

**TABLE 2:** Visual social attention results.

	% Speaker head			% Nonspeaker head		
Effect	*F*(*df*)	*F* value	*p*	*F*(*df*)	*F* value	*p*
Group	*F*(3, 84.1)	2.82	0.044	*F*(3, 88.2)	0.53	0.662
Condition	*F*(1, 1585.9)	21.26	<0.0001	*F*(1, 1585.2)	4.00	0.046
Nonverbal IQ	*F*(1, 77.2)	9.35	0.003	*F*(1, 75.1)	0.29	0.595
Age	*F*(1, 74.2)	0.00	0.965	*F*(1, 71.4)	2.32	0.132
Group × condition	*F*(3, 1584.3)	1.25	0.291	*F*(3, 1583.1)	1.43	0.232

	% Extraneous			Number transitions		
Effect	*F*(*df*)	*F* value	*p*	*F*(*df*)	*F* value	*p*
Group	*F*(3, 78.5)	1.63	0.19	*F*(3, 87.8)	2.48	0.066
Condition	*F*(1, 1583.8)	17.08	<0.0001	*F*(1, 1586.2)	0.89	0.346
Nonverbal IQ	*F*(1, 74.6)	6.40	0.014	*F*(1, 73.2)	0.98	0.325
Age	*F*(1, 72.7)	0.72	0.399	*F*(1, 76.9)	0.98	0.325
Group × condition	*F*(3, 1582.7)	2.14	0.093	*F*(3, 1584.2)	3.84	0.009

	% Valid data		
Effect	*F*(*df*)	*F* value	*p*
Group	*F*(3, 69.1)	0.92	0.434
Condition	*F*(1, 1573.7)	24.00	<0.0001
Nonverbal IQ	*F*(1, 64.5)	17.59	<0.0001
Age	*F*(1, 62.4)	0.53	0.468
Group × condition	*F*(3, 1572.1)	8.24	<0.0001

*Note*: Linear mixed effect model omnibus statistics are reported separately for spatial regions of interest (i.e., percent % time spent in area) and the number of gaze transitions, normed based upon the duration of valid data.

Proportion of valid data was greater than 90% for all groups except SCN2A (88.8%). A group by condition interaction *p* < 0.0001 indicated that there was less valid data for DYRK1A during the conversation than dyadic bid condition.

Participants looked 10.7% more to the speaker's head during the dyadic bid relative to conversation condition, *p* < 0.0001. The SCN2A group was the only group to not exhibit this effect; post hoc analysis confirmed that 4/5 SCN2A participants had no condition effects, *p* > 0.052, including one participant who never looked to the speaker's head. A main effect of group was observed, *p* = 0.037, but only a trend was observed following Bonferroni correction with iASD looking less to the speaker's head than the NT group, *p* = 0.050.

In contrast, during the conversation (relative to the dyadic bid condition), participants looked 3.42% more to nonspeakers' heads, *p* = 0.046, and 7.48% more to extraneous areas of the scene, *p* < 0.0001.

Lastly, an interaction between group and condition, *p* = 0.0092, indicated that DYRK1A was the only group to exhibit more gaze transitions during conversation than dyadic bid (*p* = 0.0014) and that DYRK1A participants exhibited more gaze transitions during the conversation condition relative to iASD (*p* = 0.0013) and NT (*p* = 0.0014).

### Auditory attention

**FIGURE 3:**
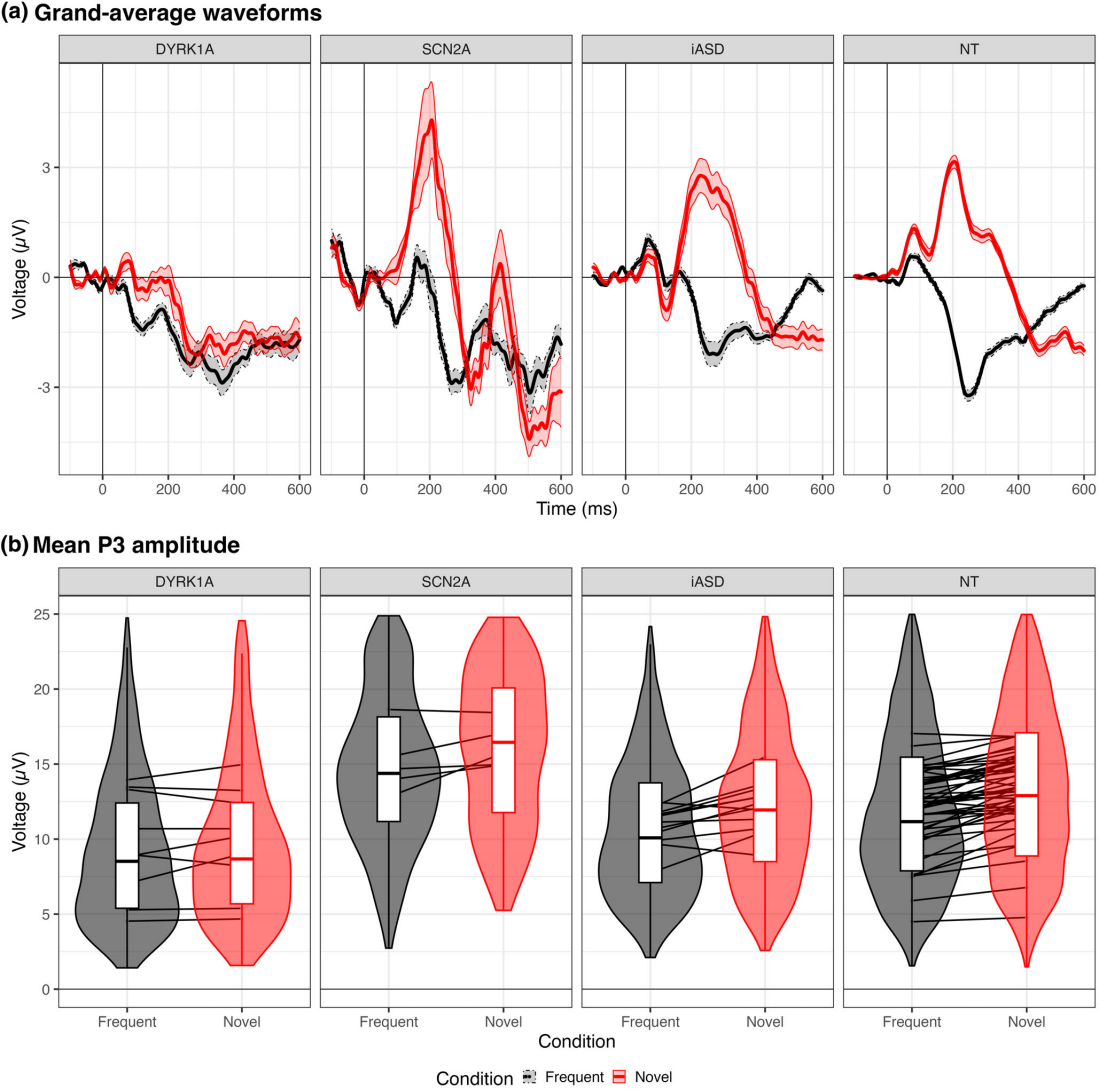
P3a amplitude during auditory attention electroencephalography experiment. (a) Grand‐average waveforms are plotted for both novel (red, solid lines) and frequent (black, dashed links) conditions for 9 DYRK1A, 5 SCN2A, 12 iASD, and 49 NT participants. (b) Group distributions are presented as violin plots to reflect group density and boxplots to indicate median (horizontal line in box) and inner quartiles (box). Average P3a scores are indicated with lines to highlight condition differences for each individual. iASD, idiopathic autism spectrum disorder; NT, neurotypical development.

EEG grand‐averaged waveforms and group‐mean P3a amplitude are presented in Figure 3. P3a amplitude was modulated by age, *F*(1,1) = 22.55, *p* < 0.0001, but not nonverbal IQ, *F*(1,1) = 0.78, *p* = 0.38. The predicted condition effect was observed, *F*(1,1) = 70.29, *p* < 0.0001, with 1.11 μV more positive P3a amplitude to novel than frequent sounds. Overall, the DYRK1A group had less positive P3a amplitudes relative to SCN2A (*p* = 0.0001) and trends relative to iASD (*p* = 0.15) and NT (*p* = 0.058), as indicated by a main effect of group, *F*(1,3) = 8.48, *p* < 0.0001. Both effects were modulated by a group by condition interaction, *F*(1,3) = 4.38, *p* = 0.0044, that indicated strong condition effects for NT, iASD, and SCN2A groups (*p* < 0.027) but not DYRK1A (*p* = 1.0).

### Exploratory characterization of alignment across task and association with individual difference measures

**FIGURE 4:**
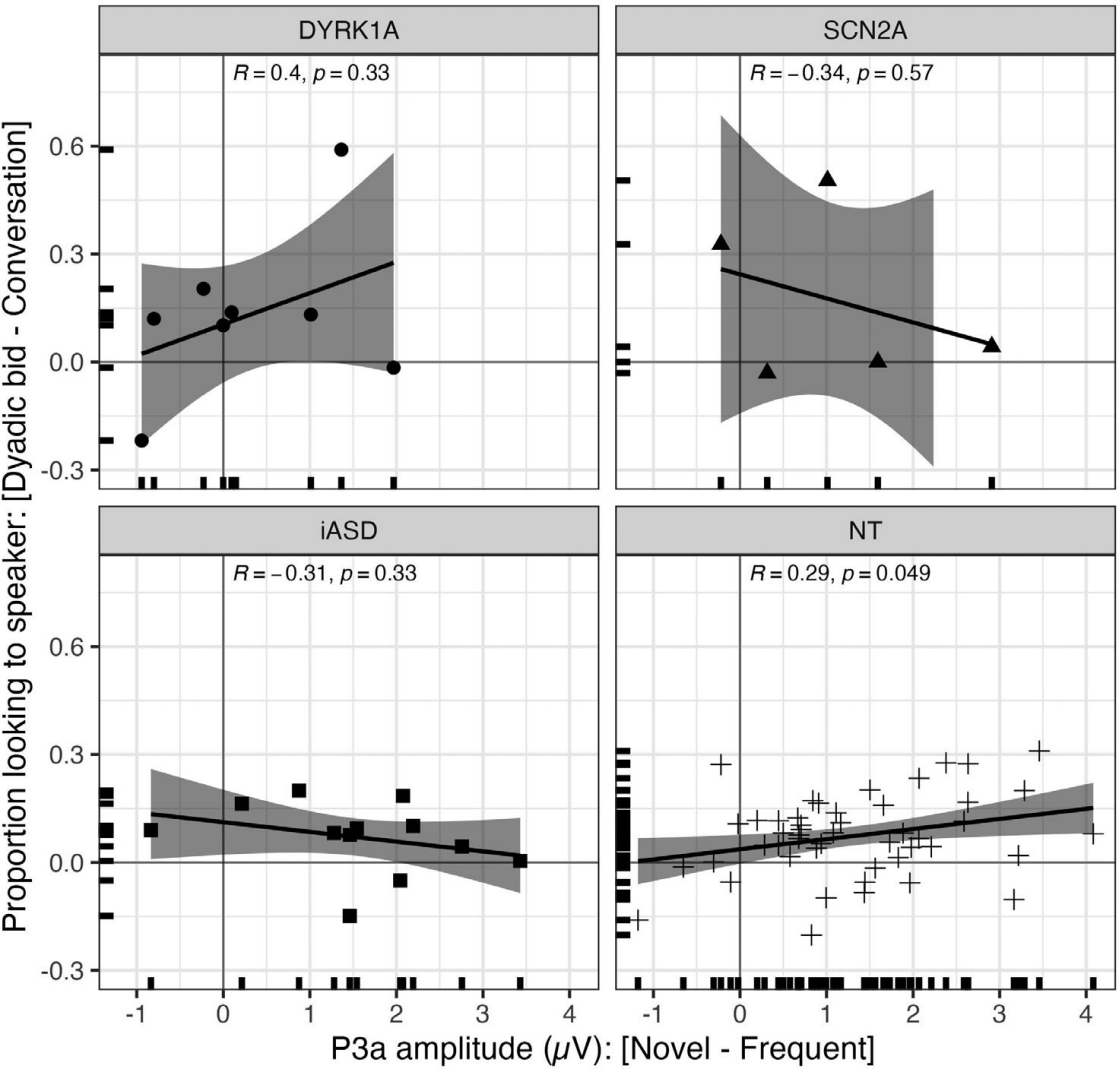
Alignment of attention measures: Positive *y*‐values indicate the predicted eye‐tracking effect (dyadic bid > conversation). Positive *x*‐values indicate the predicted electroencephalography effect (novel > frequent). Markers indicate individuals, lines indicate the correlation coefficient, and shading reflects the confidence interval of the correlation (90% interval). Rugs (i.e., marks along the axes) are included to emphasize the distribution of the data. Pearson correlation coefficients and significance (noncorrected for multiple comparisons) are included to aide descriptive characterization for each group. iASD, idiopathic autism spectrum disorder; NT, neurotypical development.

Illustration of condition differences for each experiment (visual attention via eye tracking, auditory attention via EEG) are presented in Figure 4. To better illustrate person‐level differences of associated conditions, Figures S1 and S2 graphically represent the EEG condition difference separately for each eye‐tracking condition. For the neurotypical control group, most participants (36/49, 73.4%) demonstrated increased visual attention effects (dyadic bid > conversation) corresponded with increased auditory novelty effects (novel > frequent). A similar pattern was observed in iASD (8/12, 75%), but weaker indication of alignment was observed for DYRK1A (3/8, 42.6%) and SCN2A (2/5, 40%).

Lastly, as a post hoc exploratory analysis, we examined relationships between attention (each condition and task) and individual difference factors: cognitive performance (NVIQ), age, autism severity (ADOS‐2 CSS score), and adaptive abilities (VABS‐2 composite score). Pearson correlations (false discovery rate corrected to account for multiple tests) were conducted on 21 participants with complete data. These preliminary results indicated that increased visual attention to the speaker's head was related to increased cognitive scores (conversation: *r*(19) = 0.64, *p* = 0.009; dyadic bid: *r*(19) = 0.54, *p* = 0.043) and increased adaptive scores (conversation: *r*(19) = 0.69, *p* = 0.003; dyadic bid: *r*(19) = 0.60, *p* = 0.017). Auditory attention condition effects (novel > frequent) increased with age, *r*(19) = 0.58, *p* = 0.022.

## DISCUSSION

This preliminary study is the first of its kind to examine empirical markers of attention in rare monogenetic populations with ASD and/or noted autistic features and most with intellectual disabilities and/or global developmental delay. Although it is premature to consider these specific “biomarkers” of ASD considering the lack of association with autism symptoms as measured on the ADOS‐2, this work provides early, exploratory evidence toward the feasibility and use of such outcomes. Notably, DYRK1A or SCN2A genetic groups exhibited divergent phenotypes from idiopathic ASD and neurotypical comparison groups, yet in different modalities. DYRK1A exhibited diminished auditory attention condition differences, whereas SCN2A exhibited diminished visual attention condition differences during videos of social interactions. In addition, increased visual attention to social areas was also related to cognition and adaptive abilities. Considering the comparative and translational opportunities of attention, these results point to possible biomarkers that may better describe unique functional phenotypes between DYRK1A and SCN2A populations.

First, our findings of a lack of auditory attention condition differences in DYRK1A align with prior reports of delays following acoustic startle in Dyrk1a haploinsufficient mice (Fotaki et al., 2002). Others have suggested that a vestibular processing difference may be a central mechanism of the Dyrk1a adult model (Martí et al., 2003) though there is a clear need to examine developmental processes in both animal models and patients (Arbones et al., 2019; Bon et al., 2016). For instance, recent work implicates the trajectories of macroglial cells within the corpus callosum as a potential developmental mechanism, specifically describing fewer myelinated axons that are proposed to slow action potential propagation (Pijuan et al., 2022). Taken together, one hypothesis would be that atypical attention in DYRK1A may be driven by atypical mechanisms within the central nervous system. However, considering the role of these systems in supporting visual attention, this theory would not explain intact visual attention condition differences observed for DYRK1A in this study. One possibility is that decreased auditory sensitivity could correspond to greater visual demands (i.e., DYRK1A individuals process the information in scenes more intensely on a visual domain), potentially explaining why the DYRK1A exhibited more gaze transitions during conversation. It may also be important to note that even though the measure is similar, there may be different mechanisms in DYRK1A relative to both idiopathic ASD and/or neurotypical development, which can be helpful when considering treatment decisions to bolster social skills (Simmons et al., 2019).

Second, SCN2A participants exhibited strong auditory attention condition differences, similar to that of both iASD and NT groups; yet in contrast, they showed a lack of visual attention condition differences. One possible reason may be due to atypicalities within the visual system. There is a history of atypical vision in SCN2A, including developmental eye conditions and cortical visual impairment (Berg et al., 2021; Reynolds et al., 2020). Midbrain systems (e.g., superior colliculus) are known to modulate and evoke gaze shifts in response to sensory information, including auditory stimuli, in mice (Zahler et al., 2021), and may serve as a possible mechanism of atypical attention in SCN2A. For instance, human SCN2A vestibulo‐ocular reflexes are elevated and mirror phenotypes observed in haploinsufficient Scn2a^+/−^ mice (Wang et al., 2023); importantly, authors also find that the upregulation of Scn2a expression using CRISPR‐a can rescue the vestibular responses. Considering the multitude of evidence toward atypical visual processing in SCN2A accompanied by intact auditory attention, it may be helpful to measure auditory and visual attention simultaneously and investigate mechanisms of sensory gating to better disentangle the subcortical mechanisms.

Although it is helpful to have both visual and social attention phenotypes, a primary limitation with the current study is that these tasks elicit different neural systems (i.e., auditory system ➔ general attention; visual system ➔ social attention). There is some alignment within the attention systems: Both integrate peripheral sensory signals within the central nervous system with subcortical and cortical projections playing critical developmental roles (Braddick & Atkinson, 2011; Moore & Linthicum, 2007). Additionally, there is some alignment suggesting that visual and auditory domains produce similar attention outcomes within EEG tasks (Alho et al., 1994; Sharova et al., 2009), particularly in older individuals (Günther et al., 2014), consistent with our results indicating that auditory effects increased with age. However, as noted for the SCN2A monogenic group, it is difficult to disentangle from our paradigms whether the function of each underlying neural sensory system confounds the attention markers. In other words, we cannot confirm whether or how underlying functional or structural differences in the vision or auditory systems may influence our results. We hope our findings provide a foundation for follow‐up studies to systematically evaluate the role of attention (both general and social) within each domain and potentially within a context of multisensory processing.

There are several other critical limitations to acknowledge. First, the sample size is limited for the monogenic subgroups. However, these are ultra‐rare populations (630 DYRK1A families connected with the DYRK1A Syndrome International Association^1^ and only 276 published SCN2A cases [Sanders et al., 2018]). The small iASD group also may drastically limit statistical capacity to distinguish group differences. To acknowledge this problem, where possible, we illustrated individual condition differences to highlight the variability within each group and utilized linear mixed effects statistical methods that can account for persons. Yet, efforts toward precision medicine and treatment necessitate a much larger sample size to validate biomarkers. Second, we acknowledge that it would have been helpful to have a comparison group that is cognitively matched to the monogenic groups to better differentiate the role of cognition on the attention markers. Lastly, additional candidate biomarkers should be considered. For example, examining early and late ERP components could speak to different stages of attention (e.g., N1 detection, P3 orientation, late waves as ongoing encoding). Together, we are continuing to collect data to address these limitations and explore other possible mechanisms that may be apparent as sample size increases and additional comparison groups are possible.

Although we caution against overinterpretation due to small samples, to our knowledge, this is the first investigation of neurobiological alignment of implicit visual attention and auditory attention using EEG and eye tracking. Considering that alignment was observed in the idiopathic ASD and neurotypical groups, it may be valuable to continue to evaluate attention across stimulus modalities and measurement tools in rare genetic populations. Our results emphasize the need to target not just one, but a potential combination of biomarkers, which are becoming more common in medical science (e.g., Han et al., 2015) and could be utilized in the diagnosis and treatment of neurodevelopmental disorders.

## FUNDING INFORMATION

This work was supported by the National Institutes of Health (R01 MH100047 to R.A.B., R01 MH101221 to E.E.E.) and by FamiliesSCN2A Foundation (Hudac, 2019 Action Potential Grant).

## CONFLICT OF INTEREST STATEMENT

E.E.E. is an investigator of the Howard Hughes Medical Institute.

## ETHICS STATEMENT

The University of Washington ethical review boards approved all projects and procedures. All caregivers gave written informed consent and when developmentally appropriate, participants gave written informed assent.

## Supporting information


**Data S1.** Supporting Information.

## Data Availability

The data that support the findings of this study are available on request from the corresponding author. The data are not publicly available due to privacy or ethical restrictions.

## Footnotes

^1^
Accessed via http://www.dyrk1a.org/families on January 10, 2024; as updated November 2022.
