# Supplementary material for: Visual and auditory attention in individuals with DYRK1A and SCN2A disruptive variants
Source: Autism Res. 2024 Jul 30;18(5):909–21. doi: 10.1002/aur.3202 (PMC11779982; doi:10.1002/aur.3202)

**Supplemental Material.**

**Eye tracking trends:**

An interaction trend, *p* = .093, indicated that looking more to extraneous areas during conversation was significant for *DYRK1A* (11.1%; *p* = .0029) and iASD (5.73%; *p* = .038) but marginal for *SCN2A* (10.5%; *p* = .053) and NT (2.63%; *p* = .053).

**Supplemental Table 1. Genetic and individual characterization information.** Abbreviations: ASD, autism spectrum disorder; ID, intellectual disability; GDD, global developmental delay.

|  | **gene** | **Inheritance** | **HGVSc** | **HGVSp** | **Consequence** | **ASD**  **Diagnosis** | **ID or GDD Diagnosis** | **Age of seizure**  **onset in months** |
| --- | --- | --- | --- | --- | --- | --- | --- | --- |
| 1 | *SCN2A* | de novo | c.2877C>A | p.Cys959Ter |  | yes | yes | No known  seizures |
| 2 | *SCN2A* | de novo | c.5339G>T | p.S1780I |  | yes | yes | 13 |
| 3 | *SCN2A* | de novo | c.4591C>T | p.Gln1531* | Stop-gained | yes | yes | 21 |
| 4 | *SCN2A* | unknown | c.4996C>T | p.Leu1666Phe | Missense variant | yes | yes | 111 |
| 5 | *SCN2A* | de novo | c.3296dup | p.Ser1100* |  | yes | no | Seizures with  unknown onset |
| 1 | *DYRK1A* | de novo | c.691C>T | p.Arg231Ter | Stop-gained | yes | yes | 84 |
| 2 | *DYRK1A* | unknown | c.763C>T | p.Arg255Ter | Stop-gained | yes | yes | 72 |
| 3 | *DYRK1A* | de novo | c.349C>T | p.Arg117Ter | Stop-gained | yes | yes | 36 |
| 4 | *DYRK1A* | unknown | c.1405del | p.Gln469AsnfsTer123 | Frameshift variant | yes | yes | No known  seizures |
| 5 | *DYRK1A* | unknown | c.1298dup | p.Pro434ThrfsTer15 | Frameshift variant | no | yes | 3 |
| 6 | *DYRK1A* | de novo | c.705_707delinsAC | p.Cys235Ter | Stop-gained | yes | yes | 66 |
| 7 | *DYRK1A* | unknown | c.511G>A | p.Gly171Arg | Missense variant  (CADD score v1.3=32) | yes | yes | 192 |
| 8 | *DYRK1A* | de novo | c.657C>A | p.Tyr219Ter | Stop-gained | yes | yes | 24 |

**SI Figure 1. Alignment between auditory attention via EEG and visual attention via eye tracking during the Conversation condition**: Positive y-values indicate the proportion of looking to the speaker’s head. The x-axis reflects the individual mean P3a amplitude for novel (red, triangle) and frequent (black, circle) conditions. Vertical lines are presented to aid in representing effect size between conditions for each individual. Pearson correlation values are presented and group level correlations are drawn as thick lines for novel (top correlation, red, solid) and frequent (bottom correlation black, dot-dashed) conditions. Rugs (i.e., marks along the axes) are included to emphasize the distribution of the data. Pearson correlation coefficients and significance (non-corrected for multiple comparisons) are included to aide descriptive characterization for each group. Abbreviations: iASD, idiopathic autism spectrum disorder; NT, neurotypical development.

**
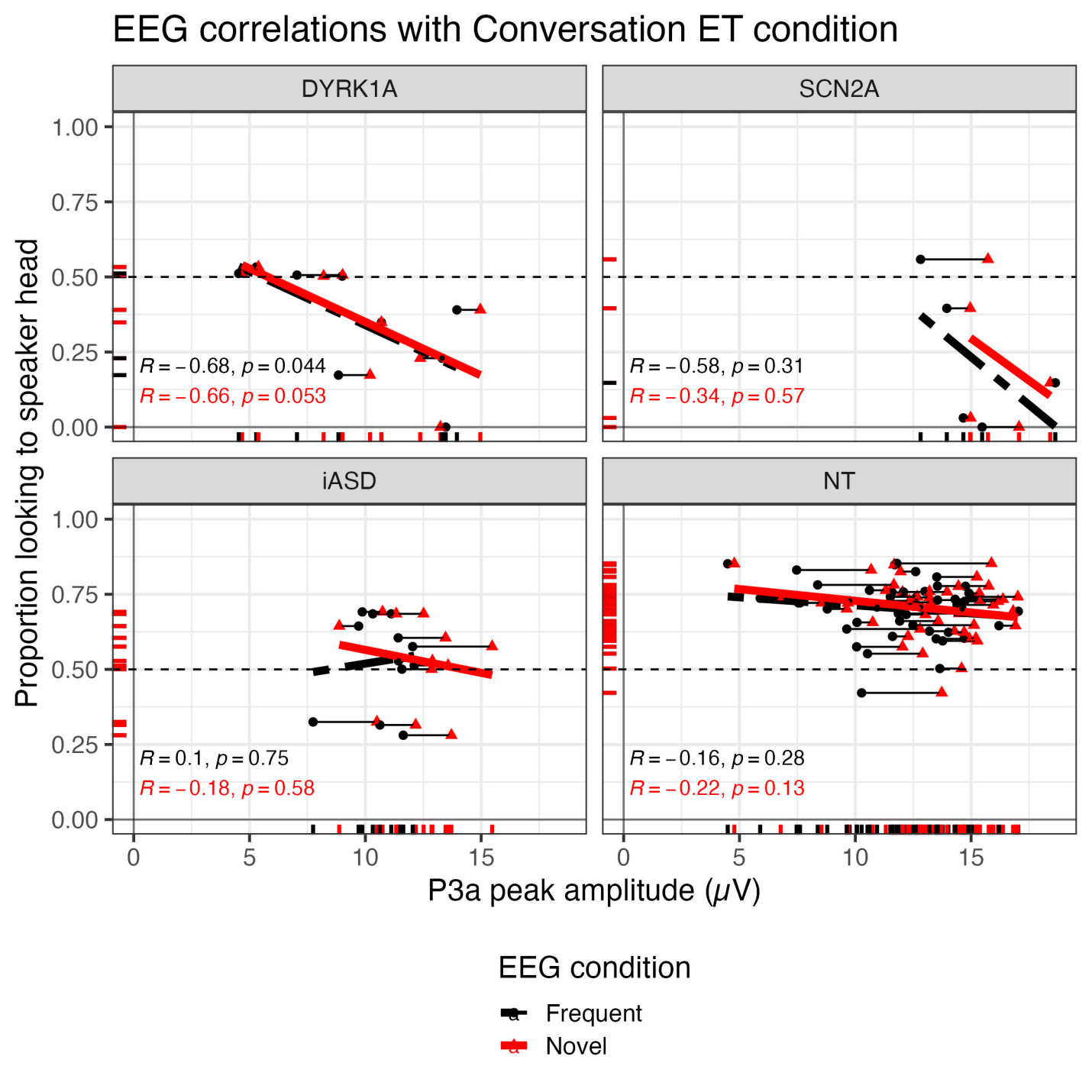
**

**SI Figure 2. Alignment between auditory attention via EEG and visual attention via eye tracking during the Dyadic Bid condition**: Positive y-values indicate the proportion of looking to the speaker’s head. The x-axis reflects the individual mean P3a amplitude for novel (red, triangle) and frequent (black, circle) conditions. Vertical lines are presented to aid in representing effect size between conditions for each individual. Pearson correlation values are presented and group level correlations are drawn as thick lines for novel (top correlation, red, solid) and frequent (bottom correlation black, dot-dashed) conditions. Rugs (i.e., marks along the axes) are included to emphasize the distribution of the data. Pearson correlation coefficients and significance (non-corrected for multiple comparisons) are included to aide descriptive characterization for each group. Abbreviations: iASD, idiopathic autism spectrum disorder; NT, neurotypical development.


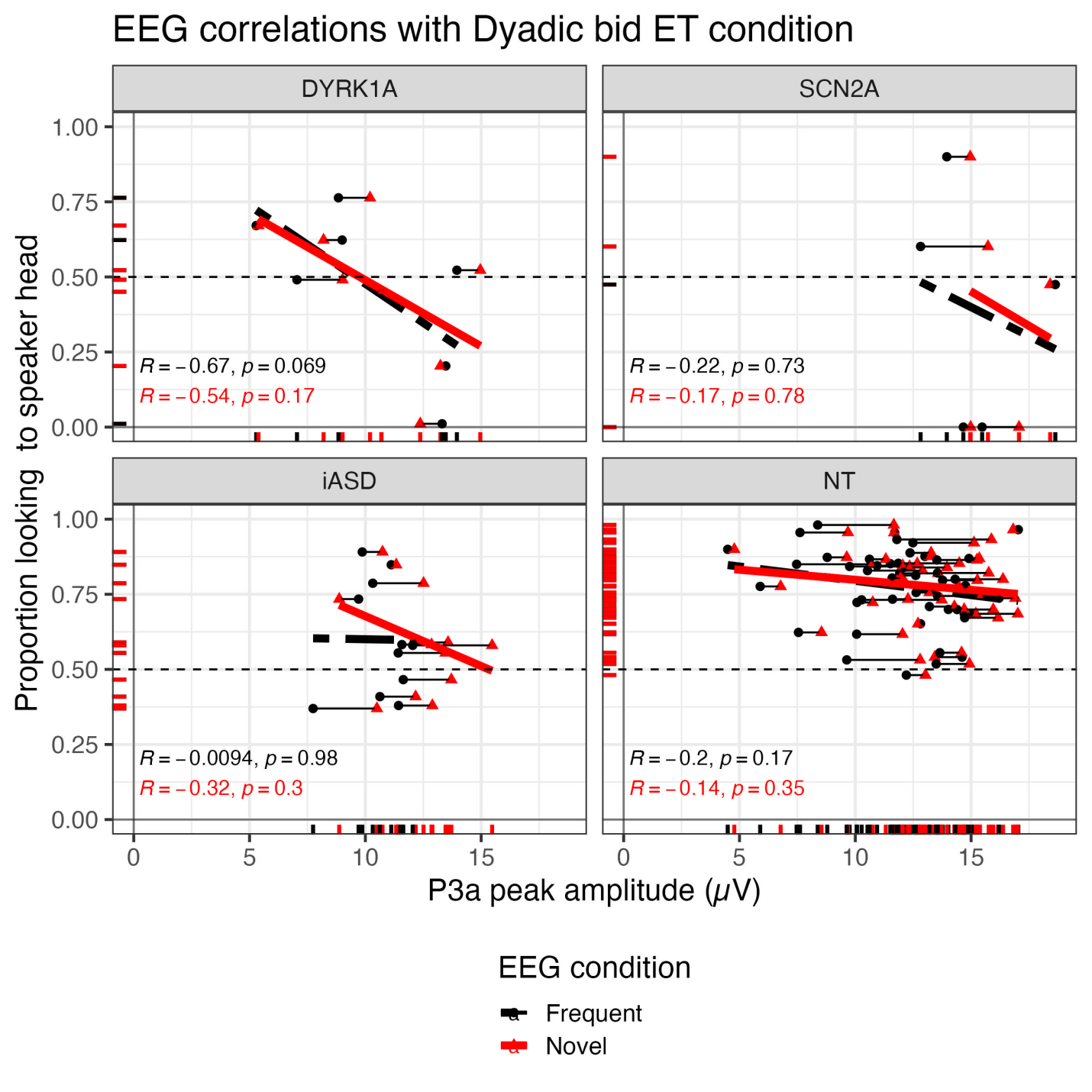

Supplement: Supplementary file 1 — Data S1. Supporting Information. [file AUR-18-909-s001.docx]
